# Physical activity in relation to health status, quality of life and compliance with World Health Organization recommendations in patients with axial spondyloarthritis

**DOI:** 10.1186/s13075-025-03575-y

**Published:** 2025-05-21

**Authors:** M. Carbo, B. Hilberdink, D. Paap, F. Wink, T. Vliet Vlieland, S. van Weely, A. Spoorenberg, S. Arends

**Affiliations:** 1 Rheumatology and Clinical Immunology, University Medical Centre Groningen, University of Groningen, Groningen, The Netherlands; 2 Orthopaedics, Rehabilitation and Physical Therapy, Leiden University Medical Center, Leiden, The Netherlands; 3 Department of physiotherapy, Saxion University of Applied Sciences, Enschede, The Netherlands; 4 Rheumatology, Medical Center Leeuwarden, Leeuwarden, The Netherlands; 5 HU University of Applied Sciences, Institute of Allied Health Professions, Utrecht, The Netherlands; 6 Rheumatology and Clinical Immunology, UMCG, P.O. Box 30001, Groningen, 9700 RB The Netherlands

**Keywords:** Physical activity, AxSpA, Quality of life

## Abstract

**Objective:**

Physical activity (PA) has well-established benefits and is a fundamental component in the management of axial spondyloarthritis (axSpA). Our objective was to evaluate (1) compliance with the World Health Organization (WHO) PA recommendations, (2) specific types and duration of PA performed by patients, and (3) association of PA with health status and quality of life (QoL) in two large Dutch cohorts of axSpA patients.

**Methods:**

In the GLAS and LUMC patient cohorts, the modified (m) and original Short QUestionnaire to ASess Health-enhancing PA (SQUASH) was used to determine fulfillment of recommendations on aerobic and muscle-strengthening PA. Univariable and multivariable linear regressions were used to analyze PA in relation to health status (ASAS-HI) and QoL (ASQoL).

**Results:**

In the GLAS (*n* = 148) and LUMC (*n* = 193) cohorts, patients were 49 ± 13 and 56 ± 14 years old, time since diagnosis was median 11 (IQR 5–21) and 23 (IQR 8–35) years and 59% and 69% were male, respectively. In total, 72% and 77% patients fulfilled the aerobic component, 40% and 36% the muscle-strengthening component and 37% and 34% both components of the WHO PA recommendations. Walking, cycling and gym or aquatic exercises were done most often. Higher (m)SQUASH score was associated with better outcome in disease-related health status (ASAS-HI) and QoL (ASQoL), also after adjusting for age, sex, BMI, disease activity and physical function.

**Conclusion:**

The minority of axSpA patients fulfilled the WHO PA recommendations. Patients were less likely to meet the muscle strengthening component than the aerobic component. A higher level of PA was associated with better disease-related health status and QoL.

## Significance and innovations

Little over a third of axSpA patients fulfilled the WHO PA recommendations. Patients were less likely to meet the muscle strengthening component than the aerobic component of the recommendations.

Walking and cycling were often done by axSpA patients and the most frequently performed sports were gym exercises and aquatic exercises.

A higher level of PA was associated with better disease-related health status and quality of life in axSpA patients.

## Introduction

Axial spondyloarthritis (axSpA) is a chronic inflammatory rheumatic disease characterized by pain and stiffness in the sacroiliac joints, often starting in the third decade of life. Treatment aims to reduce pain and physical impairment and to optimize overall functioning and health status [1]. Physical activity (PA) is beneficial for many health outcomes, including alleviating the symptoms of axSpA. PA has shown positive effects on function, spinal mobility, and pain in axSpA [2]. PA encompasses all bodily movements generated by skeletal muscles that lead to energy expenditure [3]. In the general population, there is overwhelming evidence supporting the health benefits of PA, with a 20–30% reduction in the risk of premature mortality and a decreased risk for a multitude of chronic diseases. AxSpA patients face an increased cardiovascular risk; therefore, it is even more crucial to focus on strategies that reduce these risks, including promoting PA [4, 5].

Exercise is a specific type of PA that is intentionally planned, repetitive, with the goal of improving or maintaining physical fitness [3]. The World Health Organization (WHO) recommends that individuals engage in regular exercise based on their capabilities, encompassing both aerobic and strengthening exercises targeting at least moderate intensity [6]. The 2021 European Alliance of Associations for Rheumatology (EULAR) recommendations regarding lifestyle behaviors state that these WHO PA recommendations also apply for people with rheumatic and musculoskeletal diseases (RMDs) and that patients with axSpA should be especially encouraged to exercise, as it is particularly beneficial for disease-related outcome [7]. Therefore, axSpA patients should aim for the minimum amount of physical activity recommended for all adults, constituting at least 150–300 min of moderate-intensity aerobic physical activity, or 75–150 min of vigorous-intensity aerobic physical activity per week, or a mix of both intensity levels across the weekly schedule [8]. Additionally, muscle-strengthening activities involving major muscle groups should be performed at least twice a week. Physical activity in this context incorporates various daily domains such as commuting activities (e.g., walking or cycling), work or school/study-related activities, household chores, leisure activities, as well as sports and exercise.

Previous research showed that patients with axSpA have lower PA levels and perform less vigorous intensity PA than age- and sex-matched controls from the general population [9–11]. In populations from Scandinavia and Asia, it has been shown that around 75% of axSpA fulfilled the aerobe component of the WHO PA recommendations [12]. To our knowledge, there is no study assessing the muscle-strengthening component in axSpA. However, it has been demonstrated that patients with axSpA had reduced physical performance and lower strength compared to the healthy controls [13].

There is limited data available on the type, duration and frequency of leisure time PA performed by patients with axSpA. Studies reported that bicycling, walking, hiking, back exercises, strength exercises and swimming most often performed in their cohort of axSpA patients [14–16]. As expected, local differences were found, for example cross country skiing being popular in Norway. Regarding the work-related PA, it is known that axSpA patients have impaired work participation [17]. Regular PA showed a positive effect on disease activity, physical function and pain [18]. Aerobic capacity was significantly associated with better Quality of Life (QoL) in a cross-sectional study of axSpA patients [11]. The WHO recommendations for PA takes into account moderate and vigorous intense PA, but does not include light PA. The opposite of being physically active (including light PA) is being sedentary. Sedentary behavior was found to be associated with poor QoL, [19] which aligns with the WHO recommendation to limit the amount of time spent being sedentary [8].

In order to gain insight into the type, duration, frequency and the impact of PA in axSpA, we analyzed data from two large Dutch cohorts of patients with axSpA. Our objective was to evaluate (1) compliance with the World Health Organization (WHO) PA recommendations, (2) specific types and duration of PA performed by patients, and (3) association of PA with health status and quality of life (QoL) in two large Dutch cohorts of axSpA patients.

## Methods

### Patients and setting

The participants in this study were recruited from the Groningen Leeuwarden Axial Spondyloarthritis (GLAS) cohort in the northern region of the Netherlands and from a survey study performed in three hospitals in the south-western region of the Netherlands, further referred to as the ‘LUMC cohort’. GLAS is an ongoing prospective longitudinal observational cohort study with standardized follow-up of axSpA patients visiting the outpatient clinics of the University Medical Centre Groningen (UMCG) and Medical Centre Leeuwarden (MCL). The GLAS cohort consists of consecutive axSpA patients from these tertiary and secondary referral centers who were willing to participate in the standardized follow-up [20]. In 2018, as part of the modified Short QUestionnaire to ASsess Health-enhancing physical activity mSQUASH) validation study, an axSpA-specific PA questionnaire, the mSQUASH, was added to the GLAS assessment protocol and filled-out by all patients during their outpatient visits [20, 21]. These data were used for the present analysis. In 2015, registers of three hospitals (Leiden University Medical Center (LUMC), Haga Hospital and Reinier de Graaf Gasthuis (RdGG)) were screened for axSpA patients who had ever visited the Rheumatology outpatient clinic [22]. A postal survey including a PA questionnaire, the original SQUASH, was sent to all identified patients, with a response rate of 45%. Both cohorts included a university hospital as well as one or two local hospitals. In both regions, there are also other hospitals providing care for axSpA patients. All included patients were 18 years or older and fulfilled the 1984 modified New York criteria for AS or the 2009 Assessment of SpondyloArthritis international Society (ASAS) criteria for axSpA [22, 23]. The GLAS cohort and LUMC studies were approved by the local ethics committees of all participating centers. All patients provided written informed consent according to the Declaration of Helsinki.

### Measuring PA

In order to assess PA, the mSQUASH was used in the GLAS cohort and the original SQUASH in the LUMC patients [24]. Both questionnaires measure PA during an average week in the past month and categorize PA into 4 domains: commuting activities, work or school/study activities, household activities, and leisure activities, sports and exercise. For each activity, the patient fills out: the question is not applicable or the number of days per week the activity was performed, the average time in hours and minutes spent on the activity and how physically demanding the activity was (slow/light, moderate and fast/high). The type of sport is an open-ended question, with the same structure for time and demand.

The modification of the original SQUASH resulting in the mSQUASH included adding the ‘not applicable’ option to reduce missing values, uniformization of the response options, adding the commuting activities to/from other destinations, reformulation of the questions about work/school, modernization and update of the examples for household activities and sports/exercise including adding physical therapy, adding shopping as leisure activity, changes in the questionnaire lay-out to serve feasibility and changes in the syntax, e.g. removing the standardized increase of MET value for people over 55 years of age and reducing the MET value for cycling in commuting and leisure activities [21]. For the present analysis, the syntax of the mSQUASH was also used for the SQUASH data to serve comparability [25]. The mSQUASH provides a continuous score with no fixed scoring range, a higher score represents a higher level of physical activity. The total score can be calculated by multiplying minutes per week of performing PA with a demand factor. The demand factor is based on the perceived physical demand by the patient and the metabolic equivalent of task (MET). The Ainsworth compendium was used to determine the MET value, [26] which is a manner to measure body’s expenditure of energy, and 1 MET is defined as 1 kcal/kg/hour. (5) According to the original validation protocol of the mSQUASH, data were excluded if the total minutes of activity per week exceeded 6720 (112 h), based on the assumption that respondents sleep or are inactive for at least 8 h per 24 h.

The aerobic component of the WHO PA recommendations was fulfilled if patients engaged in ≥ 150 min of moderate-intensity PA, ≥ 75 min of vigorous-intensity aerobic PA, or a combination of both. Moderate-intensity PA leads to an increased heart rate and accelerated breathing, while talking remains possible. Vigorous-intensity PA leads to increased heart rate, rapid breathing and sweating [8]. For aerobic PA, all activities in the (m)SQUASH were scored except for regular work, light to moderate household activities and shopping. For the muscle strengthening component of the WHO PA recommendations, only the sport activities were taken into account. Sport activities were classified as muscle strengthening if they increase skeletal muscle strength, power, endurance, (e.g. fitness). The muscle strengthening component was fulfilled if patients engaged in muscle strengthening activities that involve two major muscle groups, at moderate or greater intensity, and at least two days a week. To assess whether patients met the muscle-strengthening component criteria, MC (rheumatologist) and BH (physical therapist) evaluated all sports and activities, scoring them positively when the activity increase skeletal muscle strength, power, endurance, and mass in at least 2 of the major muscle groups, include the legs, back, abdomen, chest, shoulders and arms [8].

### Other clinical assessments

Clinical data from the patients in the GLAS cohort were obtained from the database at the same visit as the mSQUASH. This routinely included age, sex, time since diagnosis (years), HLA-B27 + status, body mass index (BMI), medication use, Axial Spondyloarthritis Disease Activity Score (ASDAS), Bath Ankylosing Spondylitis (AS) Disease Activity Index (BASDAI, range 0–10), C-Reactive Protein (CRP), Bath AS Functional Index (BASFI, range 0–10), and AS QoL (ASQoL, range 0–18). In all these assessments, a higher score represents higher disease activity or worse outcome.

The patient survey sent to the patient from the LUMC included age, sex, year of diagnosis and medication used in categories (among other DMARDs).

The ASAS Health Index (ASAS-HI, range 0–17) was used to determine overall functioning and health, further summarized as health status [27]. In the GLAS cohort, the ASAS-HI was available in only a subgroup of the patients. For the remaining patients, a crosswalk analyses of ASQoL data was used to calculate ´expected ASAS-HI´ scores, which was performed based on the models of Pike et al [28]. In the LUMC patients, ASAS-HI was part of the survey and, no ASQoL data were available in this group.

### Statistical analysis

Descriptive statistics were used, expressing number of patients (%), mean ± SD and median (interquartile range) for categorical, normally, and non-normally distributed data, respectively. Differences in characteristics between groups were assessed using the Chi-Square test, Independent Samples t-test and Mann Whitney U test were appropriate.

(m)SQUASH data was used to assess fulfillment of WHO recommendations on aerobic PA and muscle-strengthening. The (m)SQUASH total score takes into account the perceived physical demand reported by the patient. For the present analysis, only the minutes and METs were used to evaluate guideline adherence to improve comparability and adherence to the WHO guidelines.

To assess the type of performed PA, individual answers from the (m)SQUASH were used and if possible grouped into categories with similar to type of movement and intensity (MET value).

The association of PA (m)SQUASH total score with health status (ASAS-HI), QoL (ASQoL), BMI, disease activity (ASDAS) and physical function (BASFI) was explored using univariable linear regression analyses. Multivariable regression analysis was performed to correct the associations of ASAS-HI and ASQoL for age, sex, BMI, ASDAS, BASFI and NSAID or biological use as potential confounders for PA. The (m)SQUASH total score was square root transformed before entered into the equation. P-values ≤ 0.05 were considered statistically significant. Statistical analyses were performed with IBM SPSS Statistics for Windows, version 28.0 (IBM Corp., Armonk, N.Y., USA).

## Results

In total, 148 axSpA patients from the GLAS cohort and 193 axSpA patients from the LUMC were included. Patient characteristics are shown in Table 1. The GLAS patients on average were younger (49 vs. 56 years), more often female (69% vs. 59%), had shorter disease duration and used less frequently NSAIDs. Biological DMARD use, health status and the percentage of people participating in work or education were comparable between both cohorts. (Table 1)

### PA and fulfillment of the WHO guidelines

Light PA was done for median 420 min (IQR 180–630; IQR 3.0-10.5) in the GLAS cohort and 360 min (IQR 60–840; 6.0 h, IQR 1.0–14.0) in the LUMC patients. Patients in the GLAS cohort did more moderate intense PA for median of 870 min (IQR 476–635; 14.5 h, IQR 7.9) vs. 563 min (IQR 300–1050; 9.4 h, IQR 5.0-17.5) in the LUMC patients. The reported amount of minutes of vigorous intensity PA was very low in both cohorts. In total, 16% (GLAS) and 15% (LUMC) of patients performed any vigorous intensity PA. (Table 2)

The results regarding compliance with the WHO PA recommendations were very similar in both cohorts. The proportion of patients fulfilling the aerobic component of the PA guideline was 72% and 77%; for muscle-strengthening this was 40% and 36% for the GLAS and LUMC patients. Combined, 37% and 34% of axSpA patients, respectively, fulfilled the WHO PA recommendations. (Table 3)

### Type of PA performed

The (m)SQUASH has two intensity categories related to work, with a METs value of 3.0 for regular work (mostly sitting jobs) and a METs value of 4.5 METs for physical intensive work (e.g. regularly carrying heavy objects). In the GLAS cohort, 53% did regular work and 32% physical intensive active work vs. 48% and 16% in the LUMC patients. Light to moderately demanding household tasks were done by over 75% of patients in both cohorts, but heavily demanding household tasks were done by 73% in the GLAS cohort vs. 36% in the LUMC patients. In both cohorts, walking (82% vs. 71%) and cycling (70% vs. 61%) were done very often. The most frequently performed sports were gym exercises and aquatic exercises in both cohorts. (Table 4) Supplementary Table 1 gives a detailed overview of which sports are assigned to each category in Table 4.

### Associations between PA and disease related outcome

Univariable linear regression showed a significant association between higher (m)SQUASH total score and more favorable outcomes in ASAS-HI, ASQoL, BMI, ASDAS and BASFI. As expected, the association of PA with the BASFI had the largest explained variance of 22%. Furthermore, the (m)SQUASH total score showed significant associations with both ASAS-HI and ASQoL, with explained variance of 13–17% and 19%, respectively. Associations with BMI and ASDAS were also statistically significant, but explained variance was lower (8% and 4%, respectively). When correcting for age and sex, it was noteworthy that the increase in explained variance in this multivariate model was mostly due to age, whereas sex had no or very limited added value. When correcting for age, sex, BMI, ASDAS, BASFI and medication use, the association of PA with health status and QoL remained significant and the explained variance of the model increased to 44% for the ASAS-HI and 47% for the ASQoL. (Table 5) The results were comparable when minutes of PA instead of the (m)SQUASH total score were used (data not shown).

## Discussion

In this cross-sectional analysis involving two distinct Dutch cohorts, 34–37% of the patients with axSpA met the PA recommendations outlined by the WHO for the combination of aerobic and muscle-strengthening activities. Notably, a much larger proportion of patients showed compliance to the requirements for aerobic activities compared to muscle-strengthening exercises. The most frequently reported types of PA were walking, cycling, gym-based exercises, and aquatic workouts. A higher cumulative amount of PA exhibited a significant association with better health status and QoL, also after adjusting for age, sex, BMI, disease activity and physical function.

About half of the general Dutch population fulfills the WHO PA guidelines, which is comparable to other high income Western countries [29, 30]. Over the age of 65 years, this number drops to 41% [31]. In our patients with mean age of about 50 years, only 37% and 34% fulfils both components of the PA recommendations. In the aerobic component, patients fulfilled the WHO guidelines at a similar rate compared to the general population. However, the proportion of axSpA patients fulfilling the aerobic component (72–77%) was approximately double compared to the muscle strengthening component (36–40%) [32]. In both cohorts located in different regions of the Netherlands, we observed remarkably similar results.

The relationship between the intensity of PA and health benefits has been shown to be curvilinear, with the greatest relative risk reductions for patients moving from the inactive to a more active state [32]. Though all PA is better than inactivity, there is evidence that vigorous intense exercise improves disease activity and reduces cardiovascular risk factors in axSpA patients with active disease [33]. In our patients, fulfilling the aerobic PA component was mainly based on moderate-intensity PA. Only 15–16% of axSpA patients performed vigorous-intensity PA. These findings are in line with earlier research [34]. A qualitative study showed that patients with axSpA who participated in a high-intensity exercise program experienced this in a positive way [35]. Several strategies have been shown to enhance vigorous training, including high motivation, timing in daily routine and a lack of hindering disease symptoms in axSpA patients [36].

The types of purposeful-planned exercise performed by axSpA patients were comparable to those of the general population. The four most frequently executed PA by axSpA are the same (walking, cycling and gym- and water exercise) in both axSpA patients and in the general population [37]. Walking, swimming and cycling were found to be the most often participated sports in other axSpA patient groups as well [38, 39]. These large similarities in PA performed by axSpA patients and the general population fits with the 2021 EULAR recommendations regarding lifestyle behaviors and work participation to prevent progression of rheumatic diseases. They state that the WHO recommendations for a healthy lifestyle are also applicable to people with rheumatic diseases, i.e. avoiding physical inactivity and performing both aerobic and strengthening exercises aiming for at least moderate intensity [7].

In our study, we were especially interested in the association of the PA (m)SQUASH total score with health status (ASAS-HI) and QoL (ASQoL). Regression analysis showed that patients being more physically active experienced a better health status and QoL. After correcting for age, sex, BMI, disease activity, physical function and NSAID/biological use as potential confounders, the association of PA with health status and QoL remained significant. This increased the explained variance of the multivariable model for both health status (44%) and QoL (47%). In accordance with the present results, previous studies have demonstrated that lower levels of functional ability, higher BMI and disease activity were associated with lower PA in both patients and controls [14, 40–42]. A recent study in axSpA patients from Korea also showed that more PA was associated with physical function (BASFI) and health status (ASAS-HI), independent of disease activity (ASDAS). They found this association for recreational PA, but not for total-, work- or transport-related PA [41]. A Swedish study reported a beneficial effect of specifically recreational (intentional) PA on global function in patients with axSpA [43].

The main strength of our study is that we performed the analyses in two independent Dutch cohorts, with highly comparable results. Recently, it was demonstrated that various social determinants of health at individual patient level and country level influence disease outcomes in axSpA [44]. Unfortunately, we have limited availability of direct socioeconomic data for both cohorts. However, Statistics Netherlands publishes general differences in socioeconomic status (SES) scores based on welfare, highest level of education and recent labor participation in the areas of both cohorts [45]. The GLAS cohort is located in the provinces of Groningen and Friesland, which are rural areas with a lower SES, whereas the LUMC cohort is situated in a more urban region in the province of South Holland, where the overall SES is known to be higher. Furthermore, mSQUASH provides data on work, which is one of the indicators of SES. In patients < 65 years of age, the proportion of working patients was in favor of the urban area, although not significantly different (GLAS 64% and LUMC 73%). Additionally, patients in the rural areas were doing significantly more often physically intense work (GLAS 35% and LUMC 21%), as indirect reflection of lower SES [44]. Despite the differences in SES in the corresponding regions of the two cohorts and the varying methods of data collection (outpatient clinic visits versus postal survey), the results for both cohorts were very similar, enhancing the robustness of our findings.

The main weakness of this study includes its cross-sectional design, which prevents the establishment of a causal relationship between PA and health status or QoL. We do not know whether being more active results in a better outcome, or patients who already have a better health status and QoL are able to do more PA. Another limitation is the absence of a functional balance assessment for participants over 65 years old, a key component of the WHO physical activity guidelines to prevent falls [6]. We used the Pike method to be able to transform the data from the ASQoL to the ASAS-HI in part of the patients from the GLAS cohort. Although this is a validated method, we cannot rule out this has affected the results. The study findings may not be generalizable to early stage or non-radiographic axSpA patients, as both cohorts include a large proportion of patients with established radiographic axSpA. Finally, the mSQUASH, like all PA questionnaires, carries an inherent risk of overestimating PA levels and intensity due to possible recall bias and social desirable answers [21]. Therefore, a more direct measurement method, such as accelerometers or the doubly labeled water method, can provide a more accurate and objective assessment of PA. However, the mSQUASH questionnaire is the best, practical and cost-effective alternative for large cohort studies where more invasive or resource-intensive methods are not feasible.

Earlier research showed that in order to promote the PA recommendations among axSpA patients, a multimodal intervention is necessary, which accounts for axSpA-specific barriers and facilitators of PA. Barriers to PA have shown to be fatigue, pain and problems of timing in the daily routine [46]. AxSpA patients generally believe PA is an important self-management strategy, with beneficial effects on personal health and wellbeing [47]. A Swiss study showed that 80% of axSpA patients were unaware of the increased risk of cardiovascular diseases [48]. When providing lifestyle or behavioral advice, a patient-centered approach shows the best results, meaning that the healthcare professional assists the patient in formulating their own action plans and monitoring their progress towards self-chosen goals [49]. In order to optimize exercise behavior in axSpA, a useable intervention with attention to awareness, motivation, ability and environment has been composed [50].

## Conclusion

Little over a third of axSpA patients fulfilled the WHO recommendations for PA. Patients were less likely to meet the muscle strengthening component (36–40%) compared to the aerobic component (72–77%) of the recommendations. This aerobic PA consisted mainly of moderate-intensity PA; only 15–16% of patients performed some vigorous-intensity PA. AxSpA patients engage in the same type of PA as the general population, mainly walking, cycling and gym or aquatic exercises. More PA was significantly associated with better health status and disease-related QoL, also after correcting for age, sex, BMI, disease activity, physical function and NSAID/biological use. Therefore, in daily clinical practice, greater awareness and focus on moderate-to-vigorous intensity and muscle strengthening activity is beneficial for axSpA patients.

**Table 1:** Characteristics of AxSpA patients included in the GLAS cohort and LUMC patients

	Patients GLAS (*n* = 148)	Patients LUMC (*n* = 193)	*p*-value
Age (years)	48.5 ± 13.2	55.7 ± 14.1	< 0.001*
Sex (male), n (%)	87 (59%)	133 (69%)	0.029*
Time since diagnosis (years)	11 (5–21)	23 (8–35)	< 0.001*
HLA B27+	109 (74%)	n.a.	
BMI	27.4 ± 5.4	n.a.	
ASDAS	2.1 ± 1.0	n.a.	
BASDAI (0–10)	3.9 ± 2.4	n.a.	
CRP	2.1 (1.0–5.0)	n.a.	
BASFI (0–10)	2.8 (1.1–5.7)	n.a.	
***Quality of life***			
ASQoL (0–18)	4.0 (1.0–9.0) *n* = 90	n.a.	
ASAS-HI (0–17)	4.0 (2.0-8.7) *n* = 44	5.3 (2.1-8.0) *n* = 189	0.42
ASAS HI crosswalk	4.4 (1.9–8.1) *n* = 102	n.a.	
***Medication***			
No medication use	13 (10%)	27 (14%)	0.30
NSAID use	87 (59%)	119 (62%)	0.025*
bDMARDs use	58 (39%)	74 (38%)	0.17
***Work/school***			
Current participation, n (%)	85 (57%)	106 (55%)	0.36
Hours a week	36 (29–40)	36 (20–40)	0.60

Data presented as number of patients (%), mean ± SD or median (IQR). BMI, body mass index; ASDAS, Axial Spondyloarthritis Disease Activity Score; BASDAI, Bath Ankylosing Spondylitis Disease Activity Index; CRP, C-reactive protein; BASFI, Bath Ankylosing Spondylitis Functional Index; ASQoL, Ankylosing Spondylitis Quality of Life; ASAS-HI, ASAS-Health index; NSAID, Non Steroid Anti Inflammatory Drug; bDMARDs, biologic disease-modifying antirheumatic drugs; N.a., not available. *p-value ≤ 0.05

**Table 2:** Physical activity: minutes spent in light, moderate and vigorous PA by patients with AxSpA

	GLAS (*n* = 148)	LUMC (*n* = 193)
Light PA min. (1.5-<3 METs)	420 (180–630)	360 (60–840)
Moderate PA min. (3-<6 METs )	2195 (870–2899)	1525 (590–2740)
Vigorous PA min. (≥ 6 METs)	0 (0–0)	0 (0–0)
Patients doing any (≥ 1) min of vigorous intense PA	23 (16%)	29 (15%)

Data presented as median (IQR). Data represented is PA in an average week, as assessed by the (m)SQUASH

**Table 3:** Compliance with WHO PA recommendations for adults in patients with AxSpA

WHO PA guideline	GLAS (*n* = 148)	LUMC (*n* = 193)
WHO PA guideline for adults	58 (39%)	66 (34%)
Aerobic PA component	140 (95%)	171 (89%)
Muscle strengthening component	59 (40%)	69 (36%)

Data presented as number of patients (%)

**Table 4:** Type and frequencies of physical activities performed by patients with AxSpA

Exercise types	METs	GLAS (*n* = 148)			LUMC (*n* = 193)		
		*n*	Minutes a week	Freq.	*n*	Minutes a week	Freq.
Work or school/study							
Regular	3.0	79 (53%)	1800 (1200–2160)	-	93 (48%)	1920 (1200–2400)	-
Physical intensive	4.5	47 (32%)	600 (240–1200)	-	30 (16%)	1020 (525–2400)	-
Household activities							
Light to moderate	2.5	140 (95%)	420 (210–698)	-	147 (76%)	450 (240–900)	-
Highly demanding	3.5	108 (73%)	83 (60–210)	-	69 (36%)	70 (40–180)	-
Leisure activities							
Gardening	3.0	72 (49%)	90 (60–120)	1 (0–1)	74 (38%)	90 (49–188)	1 (1–3)
Home maintenance	3.8	57 (39%)	120 (60–240)	1 (0–1)	71 (37%)	90 (45–240)	1 (1–2)
Shopping	2.5	96 (65%)	78 (60–120)	1 (1–3)	-	-	-
Commuting activities, Sports and exercise							
Walking*	3.5	122 (82%)	240 (110–510)	7 (3–10)	137 (71%)	190 (120–315)	5 (2–7)
Cycling*	5.8–10.0	104 (70%)	180 (106–420)	5 (3–8)	117 (61%)	200 (90–360)	4 (2–6)
Gym exercise	5.5–6.8	31 (21%)	180 (60–270)	2 (1–3)	34 (18%)	120 (120–195)	2 (1–3)
Aquatic exercise	3.0-5.8	20 (14%)	60 (60–113)	1 (1–2)	24 (12%)	60 (45–120)	1 (1–2)
Home exercise	3.8–4.8	15 (10%)	105 (60–165)	3 (1–7)	13 (7%)	75 (60–163)	2 (1–5)
Supervised exercise	4.3-5.0	14 (9%)	60 (30–93)	1 (1–2)	19 (10%)	135 (90–135)	1 (1–1)
Running	5.0–8.0	9 (6%)	75 (30–150)	2 (1–3)	17 (9%)	90 (40–240)	2 (1–3)
Competitive sports	4.0-7.8	7 (5%)	180 (75–360)	2 (1–3)	19 (10%)	150 (90–240)	1 (1–2)
Body and mind exercise	3.0-3.3	2 (1%)	113 (105–113)	5 (2–5)	8 (4%)	105 (64–158)	1 (1-3.5)
Other sports	3.0-10.3	5 (3%)	90 (50–300)	1 (1–3)	3 (2%)	240 (120–240)	1 (1–1)

Data presented as number of patients (%) or median (IQR)

*The same activities scored in different (m) SQUASH domains were combined. For example, cycling in commute, leisure activities and sports were grouped together (including duration and frequency)

**Table 5:** Associations of (m)SQUASH total score with health status and QoL in patients with AxSpA

	(m)SQUASH			(m)SQUASH corrected for age and sex			(m)SQUASH corrected for age, sex, BMI, ASDAS, BASFI, NSAID and biological use		
	B (95%CI)	R2	*p*-value	B (95%CI)	R2	*p*-value	B (95%CI)	R2	*p*-value
**ASAS-HI**									
GLAS	-2.9 (-4.3- -2.6)	0.17	< 0.01*	-2.8 (-4.1- -1.5)	0.22	< 0.01*	-2.8 (-5.8- -0.1)	0.44	< 0.01*
LUMC	-3.2 (-4.3- -2.0)	0.13	< 0.01*	-2.8 (-3.9- -1.6)	0.25	< 0.01*	-	-	-
**ASQoL**									
GLAS	-2.4 (-3.5- -1.4)	0.19	< 0.01*	-2.4 (-3.4- -1.3)	0.24	< 0.01*	-3.1 (-5.4- -0.7)	0.47	< 0.01*
**BMI**									
GLAS	-1.6 (-2.5- -0.7)	0.08	0.01*	-1.1 (-2.0- -0.2)	0.13	0.02*	-	-	-
**ASDAS**									
GLAS	-5.9 (-10.7- -1.1)	0.04	0.02*	-6.0 (-10.5- -1.5)	0.14	0.01*	-	-	-
**BASFI**									
GLAS	-5.4 (-7.4- -3.5)	0.22	< 0.01*	-4.5 (-6.6- -2.4)	0.26	< 0.01*	-	-	-

## Data Availability

Data are available from the University of Groningen—UMCG Institutional Data Access for researchers who meet the criteria for access to confidential data. The local ethics committees of the Medical Center Leeuwarden (MCL) and the University Medical Center Groningen (UMCG) will maintain the ethical restrictions of the data. The Data Protection Officer of the UMCG will maintain the legal restrictions and appropriate codes of conduct. Permission is required prior to access. Data requests can be sent to Research Data Office University of Groningen: researchdata@rug.nl.

## Abbreviations

AS: Ankylosing Spondylitis
ASAS: Assessment of Spondyloarthritis International Society
ASAS-HI: Assessment of Spondyloarthritis International Society Health Index
ASDAS: Axial Spondyloarthritis Disease Activity Score
ASQoL: Ankylosing Spondylitis Quality of Life
axSpA: Axial Spondyloarthritis
BASDAI: Bath Ankylosing Spondylitis Disease Activity Index
BASFI: Bath Ankylosing Spondylitis Functional Index
BMI: Body Mass Index
CRP: C-Reactive Protein
DMARD: Disease-Modifying Anti-Rheumatic Drug
EULAR: European Alliance of Associations for Rheumatology
GLAS: Groningen Leeuwarden Axial Spondyloarthritis
HLA-B27: Human Leukocyte Antigen B27
IQR: Interquartile Range
LUMC: Leiden University Medical Center
MCL: Medical Centre Leeuwarden
MET: Metabolic Equivalent of Task
mSQUASH: Modified Short Questionnaire to Assess Health-enhancing physical activity
NSAID: Non-Steroidal Anti-Inflammatory Drug
PA: Physical Activity
QoL: Quality of Life
RdGG: Reinier de Graaf Gasthuis
RMDs: Rheumatic and Musculoskeletal Diseases
SD: Standard Deviation
SQUASH: Short Questionnaire to Assess Health-enhancing physical activity
UMCG: University Medical Centre Groningen
WHO: World Health Organization
